# HbA1c as a Predictor of Complications in Anterior and Posterior Colporrhaphy

**DOI:** 10.7759/cureus.103431

**Published:** 2026-02-11

**Authors:** Alexandra McQuillen, Vaishnavi J Patel, Devki Patel, Kimberly C Toumazos, Young Son, David O Sussman

**Affiliations:** 1 Department of Research, Philadelphia College of Osteopathic Medicine, Philadelphia, USA; 2 Department of Family Medicine, The University of Texas at Austin Dell Medical School, Austin, USA; 3 Office of Research, Texas Tech University Health Sciences Center School of Medicine, Lubbock, USA; 4 Department of Urology, University of Kentucky College of Medicine, Lexington, USA; 5 Department of Urology, Jefferson Stratford Hospital, Stratford, USA; 6 Department of Urology, Jefferson Washington Township Hospital, Sewell, USA

**Keywords:** anterior and posterior colporrhaphy, colporrhaphy, diabetes mellitus type 2, glycemic control, hba1c, pelvic organ prolapse, surgical complication rates, surgical complications, type 2 diabetes mellitus, urinary tract infections

## Abstract

Importance: Colporrhaphy is a surgical option for anterior or posterior compartment vaginal prolapse. Colporrhaphy has a high complication rate. Previous literature proved that individuals diagnosed with diabetes had higher rates of complications in other urological surgeries, especially in prosthesis or mesh insertion.

Objective: This study aims to determine if HbA1c is associated with anterior or posterior colporrhaphy complications.

Study design: The 2021 National Surgical Quality Surgical Improvement database was used for a retrospective review study of patients who had anterior or posterior colporrhaphy. The cohort was subdivided into those with HbA1c ≤6.4 and those with ≥6.5. In a 30-day composite, major and minor complications were analyzed. A multivariate logistic regression was then performed to predict complications.

Results: A total of 182 patients were included, with 46 (25.3%) in the ≥6.5 cohort and 136 (74.7%) in the ≤6.4 cohort. The ≥6.5 group was older (67.9 years vs. 64.1 years), whereas there was no noted difference in race, hypertension, or previous abdominal operations. The composite complication was higher in the ≥6.5 group at 21.7% (n = 10) compared to 10.3% (n = 14) in the ≤6.4 group. The most common complication was urinary tract infection (8.2%, n = 15). On adjusted analysis, the ≥6.5 group had higher odds of complication (OR 3.16, p = 0.05).

Conclusions: Diabetes should be considered a comorbidity in patients undergoing anterior or posterior colporrhaphy. Our study shows that there are three times the odds of complications in patients with higher HbA1c. Strict glycemic control should be implemented to decrease the risk.

## Introduction

The annual incidence rate of pelvic organ prolapse (POP) in the United States is 1.5-1.8 per 1,000, resulting in an impact of more than five million women in the United States [1,2]. Colporrhaphy is the standard of care for POP, with indications of use including incontinence and/or POP. Preoperatively, diabetes mellitus (DM) is a known risk factor for POP, with a prevalence of 37.3 million people (11.3%) in the United States [3]. Of patients who will require a surgical procedure in their lifetime, up to 15% have been diagnosed with DM. Hemoglobin A1c (HbA1c) is often used to indicate glycemic control, with an ≥8-fold increase in poor surgical outcomes [4]. There is currently a paucity of literature on glycemic control and its impact on colporrhaphy outcomes, identifying a gap in research that could benefit perioperative patient outcomes [4,5]. Current literature has established a 2-to-3 fold increased risk of complication among patients undergoing urogynecologic surgery with a preoperative HbA1c of 8 or higher, elucidating the need for a more tailored outlook on specifically colporrhaphy [6]. 

As a measure of long-term glycemic control in patients with DM, the American Diabetes Association subscribes to the use of HbA1c to determine operative readiness [7]. Increased preoperative HbA1c has been shown to be a predisposing risk factor for postoperative complications including anastomotic leaks, poor wound healing, and infections [8]. In a systematic review and meta-analyses, mortality rates in diabetic patients were higher at 3.5% as compared to non-diabetic patients at 0.0%. These findings indicate that glycemic control in the perioperative period could be a focus for reduction of postoperative complications in a diversity of surgeries [9]. 

Associated factors contributing to complication rates have been minimally defined in patients undergoing anterior or posterior colporrhaphy. Risk factors for POP recurrence previously elucidated in literature include parity, vaginal delivery, BMI, age, and preoperative prolapse stage [10]. 

Our primary objective was to identify the incidence of 30-day pre, intra, and postoperative complications following anterior or posterior colporrhaphy in patients with HbA1c ≥6.5 or ≤6.4 using a national database. The study's secondary objectives were to compare the independent risks of surgical complications and to define the most common adverse events. 

## Materials and methods

Source of data

The ACS NSQIP is a database that adheres to the regulations set forth by the Health Insurance Portability and Accountability Act (HIPAA), and it contains information on patient cases from approximately 702 hospitals. The dataset consists of data from 2021 since that was the only year NSQIP recorded HbA1c data. The principal operative procedure cases are identified based on Current Procedural Terminology (CPT) codes. The program aims to evaluate the quality of care provided after surgical procedures. Since the ACS NSQIP is de-identified, this retrospective study was exempt from the institutional review board.

Population of study and analysis

**Figure 1:**
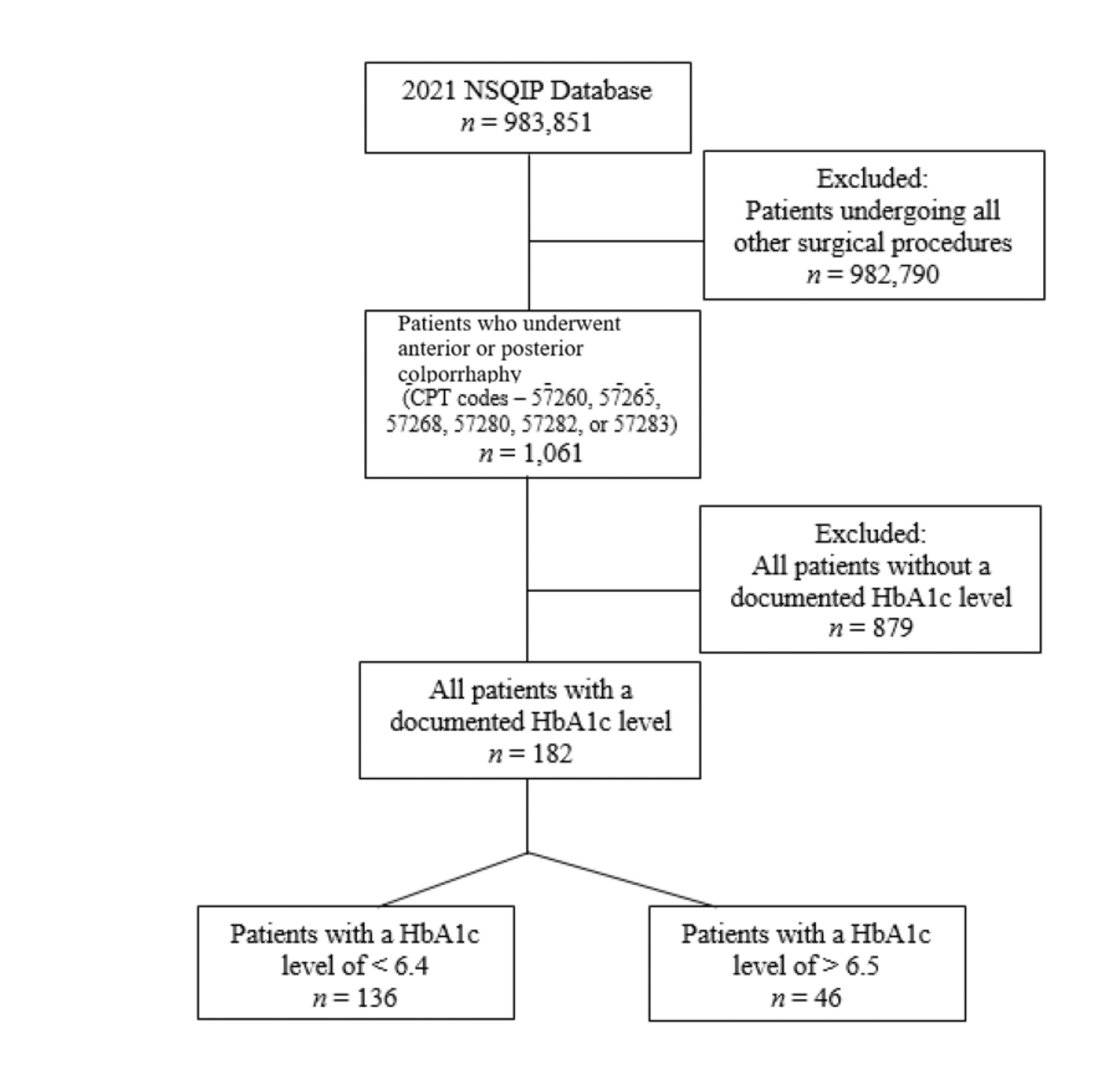
Study flow diagram

We analyzed the NSQIP database from 2021 to study patients who had undergone anterior or posterior colporrhaphy. Our inclusion criteria included procedures with primary CPT codes of 57260 (Combined anterior and posterior colporrhaphy with cystoscopy), 57265 (Combined anterior and posterior colporrhaphy with cystoscopy and neurotomy repair), 57268 (Repair of enterocele; through vaginal approach), 57280 (Colpopexy, abdominal approach), 57282 (Colpopexy, vaginal extraperitoneal approach), or 57283 (Colpopexy, vaginal intraperitoneal approach) which led to a total of 1061 patients. As seen in Figure 1, patients without a documented HbA1c level (total of 878) were excluded. After this, the cohort was divided into HbA1c ≥6.5 and HbA1c ≤6.4.

Patients were categorized using a preoperative HbA1c threshold of 6.5%, corresponding to the American Diabetes Association diagnostic criterion for diabetes mellitus. This cutoff was selected to maximize clinical interpretability at the time of surgical referral and to evaluate whether perioperative risk is detectable at the diagnostic threshold for diabetes, rather than only at higher levels of hyperglycemia.

Analysis comparing the HbA1c ≥6.5 and HbA1c ≤6.4 groups was conducted. Preoperative, perioperative, and postoperative variables were compared between the two groups. Minor complications included urinary tract infections, superficial surgical site infections (SSIs), and transfusion. Significant complications included unplanned readmission and organ space SSI. These categories were based on those in a study by Hong et al. and clinical expertise [11]. Chi-square test analysis was used for categorical variables, while the two-sample T-test or Fisher's exact test was used for continuous variables as appropriate. Logistic regression univariate and adjusted analysis was performed with peri and postoperative complications as dependent variables using HbA1c ≤6.4 as the referent. We conducted the statistical analysis using R version 4.0.2 (R Foundation for Statistical Computing, Vienna, Austria), and statistical significance was accepted at a p-value < 0.05.

## Results

Of the 182 patients who underwent colporrhaphy, 46 patients (25.3%) had an HbA1c of ≥6.5, while 136 patients (74.7%) had an HbA1c of ≤6.4. Demographics and past medical history of patients undergoing anterior/posterior colporrhaphy are shown in Table 1. HbA1c ≥6.5 patients were older (mean age 67.9 years (SD 10.5) vs. 64.1 years (SD 11.2), p = 0.045) with unknown race (56.5%, n = 26 vs. 38.2%, n = 52, p = 0.046), and more likely to be ASA class 3 (60.9%, n =28 vs. 33.8%, n = 46, p = 0.002). Patients with HbA1c ≥6.5 additionally were more likely to have insulin-dependent DM (21.7%, n = 10 vs. 2.2%, n = 3, p < 0.001) and non-insulin-dependent DM (58.7%, n = 27 vs. 18.4%, n = 25, p < 0.001). Patients with HbA1c ≥6.5 also had hypertension requiring medication at higher rates (71.7%, n = 33 vs. 44.9%, n = 61, p = 0.003). There was no significant difference in height, weight, BMI, parity, known race, discharge or admission status, or functional health status prior to surgery. Additionally, there were no differences in past medical history characteristics including COPD history, history of heart failure 30 days before surgery, bleeding disorders, prior abdominal operations, prior pelvic operations, endometriosis, prolonged urinary retention, or current smoking status. Preoperative laboratory values were also of no significance in our findings, which measured values included serum sodium, BUN, serum creatinine, platelet count, PTT, INR, WBC, and hematocrit. 

**Table 1:** Demographics and Past Medical History for Anterior and Posterior Colporrhaphy Patients p-values for categorical variables were calculated using χ² tests of independence (df = 1 for all 2×2 comparisons). χ² statistics <0.01 are displayed as <0.01 for clarity. χ² statistics and degrees of freedom are not reported for variables with no between-group variability (e.g., 100% vs 100% or 0% vs 0%), for which statistical testing is not informative. Continuous variables were compared using t-tests.

	HbA1c ≥ 6.5 n = 46	HbA1c ≤ 6.4 n = 136	Total Cohort n = 182	p-value	χ²	df
Demographics						
Age; mean (SD); years	67.9 (10.5)	64.1 (11.2)	65.1 (11.1)	0.045	-	-
Height; mean (SD); inches	62.4 (2.88)	63.2 (2.80)	63.0 (2.83)	0.104	-	-
Weight; mean (SD); pounds	162 (38.0)	166 (38.8)	165 (38.5)	0.493	-	-
BMI; mean (SD)	29.0 (5.50)	29.3 (6.34)	29.2 (6.12)	0.831	-	-
Female	46.0 (100%)	136 (100%)	182 (100%)	-	-	-
Parity						
Nulliparity	4.00 (8.70%)	14.0 (10.3%)	18.0 (9.90%)	0.98	<0.01	1
Uniparity	4.00 (8.70%)	11.0 (8.10%)	15.0 (8.20%)	1	<0.01	1
Multiparity	38.0 (82.6%)	111 (81.6%)	149 (81.9%)	1	<0.01	1
Race						
Caucasian	18.0 (39.1%)	66.0 (48.5%)	84.0 (46.2%)	0.35	0.87	1
American Indian or Alaska Native	0 (0%)	4.00 (2.90%)	4.00 (2.20%)	0.552	0.35	1
Black or African American	2.00 (4.30%)	4.00 (2.90%)	6.00 (3.30%)	1	<0.01	1
Asian	0 (0%)	7.00 (5.10%)	7.00 (3.80%)	0.26	1.27	1
Unknown/Not Reported	26.0 (56.5%)	52.0 (38.2%)	78.0 (42.9%)	0.046	3.98	1
Some Other Race	0 (0%)	3.00 (2.20%)	3.00 (1.60%)	0.729	0.12	1
Hispanic	0 (0%)	0 (0%)	0 (0%)	-	-	-
Inpatient	23.0 (50.0%)	52.0 (38.2%)	75.0 (41.2%)	0.219	1.51	1
Discharge to Home	46.0 (100%)	136 (100%)	182 (100%)	-	-	-
Functional Health Status Prior to Surgery						
Independent	46.0 (100%)	133 (97.8%)	179 (98.4%)	0.729	0.12	1
Dependent	0 (0%)	3.00 (2.20%)	3.00 (1.60%)			
ASA Classification						
Class 1	0 (0%)	7.00 (5.10%)	7.00 (3.80%)	0.26	1.27	1
Class 2	18.0 (39.1%)	77.0 (56.6%)	95.0 (52.2%)	0.06	3.54	1
Class 3	28.0 (60.9%)	46.0 (33.8%)	74.0 (40.7%)	0.002	9.33	1
Class 4	0 (0%)	5.00 (3.70%)	5.00 (2.70%)	0.425	0.64	1
Past Medical History						
Insulin-Dependent DM	10.0 (21.7%)	3.00 (2.20%)	13.0 (7.10%)	<0.001	16.94	1
Non-insulin-Dependent DM	27.0 (58.7%)	25.0 (18.4%)	52.0 (28.6%)	<0.001	25.43	1
History of Severe COPD	0 (0%)	1.00 (0.700%)	1.00 (0.500%)	1	<0.01	1
Heart Failure in 30 Days before Surgery	1.00 (2.20%)	2.00 (1.50%)	3.00 (1.60%)	1	<0.01	1
Hypertension Requiring Medication	33.0 (71.7%)	61.0 (44.9%)	94.0 (51.6%)	0.003	8.90	1
Bleeding Disorders	2.00 (4.30%)	3.00 (2.20%)	5.00 (2.70%)	0.805	0.06	1
Prior Abdominal Operations	17.0 (37.0%)	43.0 (31.6%)	60.0 (33.0%)	0.628	0.23	1
Prior Pelvic Operations	28.0 (60.9%)	91.0 (66.9%)	119 (65.4%)	0.572	0.32	1
Endometriosis	1.00 (2.20%)	1.00 (0.700%)	2.00 (1.10%)	1	<0.01	1
Prolonged Urinary Retention	1.00 (2.20%)	0 (0%)	1.00 (0.500%)	0.568	0.33	1
Current Smoker	1.00 (2.20%)	2.00 (1.50%)	3.00 (1.60%)	1	<0.01	1

Operative characteristics, admission characteristics, and individual and composite complications for each HbA1c parameter are shown in Table 2. Compared to HbA1c ≤6.4, HbA1c ≥6.5 was associated with an increased estimated probability of mortality (p = 0.012) (Table 2). 

**Table 2:** Operative Characteristics for Anterior and Posterior Colporrhaphy Cases p-values for categorical variables were calculated using χ² tests of independence (df = 1 for all 2×2 comparisons). χ² statistics <0.01 are displayed as <0.01 for clarity. χ² statistics and degrees of freedom are not reported for variables with no between-group variability (e.g., 100% vs 100% or 0% vs 0%), for which statistical testing is not informative. Continuous variables were compared using t-tests.

	HbA1c ≥ 6.5 n = 46	HbA1c ≤ 6.4 n = 136	Total Cohort n = 182	p-value	χ²	df
Anesthesia						
General Anes	42.0 (91.3%)	122 (89.7%)	164 (90.1%)	0.977	<0.01	1
MAC/IV Sedation Anes	1.00 (2.20%)	3.00 (2.20%)	4.00 (2.20%)	1	<0.01	1
Spinal Anes	3.00 (6.50%)	10.0 (7.40%)	13.0 (7.10%)	1	<0.01	1
Epidural Anes	0 (0%)	1.00 (0.700%)	1.00 (0.500%)	1	<0.01	1
Surgical Specialty						
Gynecology	44.0 (95.7%)	130 (95.6%)	174 (95.6%)	1	<0.01	1
Obstetrician-Gynecologist	18.0 (39.1%)	46.0 (33.8%)	64.0 (35.2%)	0.636	0.22	1
Urogynecologist	27.0 (58.7%)	86.0 (63.2%)	113 (62.1%)	0.709	0.14	1
Gynecologic Oncologist	1.00 (2.20%)	2.00 (1.50%)	3.00 (1.60%)	1	<0.01	1
Urology	2.00 (4.30%)	5.00 (3.70%)	7.00 (3.80%)	1	<0.01	1
Plastics	0 (0%)	1.00 (0.700%)	1.00 (0.500%)	1	<0.01	1
Concomitant Procedure	36.0 (78.3%)	108 (79.4%)	144 (79.1%)	1	<0.01	1
Estimated Probability of Mortality	0.00100	0.000810	0.000876	0.125	-	-
Estimated Probability of Morbidity	0.0805	0.0680	0.0712	0.012	-	-
Operation time; mins	126 (71.4)	128 (72.2)	127 (71.8)	0.916	-	-
Length of total hospital stay; days	0.978 (1.31)	0.743 (0.974)	0.802 (1.07)	0.197	-	-
Days from Operation to Discharge	0.978 (1.31)	0.743 (0.974)	0.802 (1.07)	0.197	-	-

Major and minor 30-day surgical complications are shown in Table 3, with no difference in superficial SSI, urinary tract infections (UTIs), bleeding transfusions, organ space SSI, or readmission among the two HbA1c cohorts. When controlling in univariate and multivariate analysis, these major and minor complications were not significantly associated with HbA1c ≥6.5 (Table 4). Multivariate analysis revealed that HbA1c of ≥6.5 was associated with 30-day perioperative adverse events following colporrhaphy (OR 3.16, 95% CI 1.00 - 10.22, p = 0.050) while controlled for age, BMI, race, ethnicity, smoking status, diabetes, COPD, steroids, and hypertension. HbA1c ≥6.5 was additionally significantly associated with 30-day perioperative adverse events in univariate analysis (OR 2.42, 95% CI 0.970 - 5.88, p = 0.052) (Table 5). 

**Table 3:** 30-Day Surgical Complications Following Anterior and Posterior Colporrhaphy p-values for categorical variables were calculated using χ² tests of independence (df = 1 for all 2×2 comparisons). χ² statistics <0.01 are displayed as <0.01 for clarity. χ² statistics and degrees of freedom are not reported for variables with no between-group variability (e.g., 100% vs 100% or 0% vs 0%), for which statistical testing is not informative. Continuous variables were compared using t-tests.

	HbA1c ≥ 6.5 n = 46	HbA1c ≤ 6.4 n = 136	Total Cohort n = 182	p-value	χ²	df
Minor Complications						
Superficial Surgical Site Infection	1.00 (2.20%)	2.00 (1.50%)	3.00 (1.60%)	1	<0.01	1
Urinary Tract Infections	5.00 (10.9%)	10.0 (7.40%)	15.0 (8.20%)	0.66	0.19	1
Bleeding Transfusions	2.00 (4.30%)	2.00 (1.50%)	4.00 (2.20%)	0.569	0.32	1
Major Complications						
Organ Space Surgical Site Infection	1.00 (2.20%)	0 (0%)	1.00 (0.500%)	0.568	0.33	1
Readmission	1.00 (2.20%)	1.00 (0.700%)	2.00 (1.10%)	1	<0.01	1

**Table 4:** 30-Day Surgical Complication Logistic Regression Analysis Following Anterior and Posterior Colporrhaphy Reference is HbA1c ≤6.4. Controls for multivariate were age, BMI, race (minority or not), ethnicity (Hispanic or not), smoking status, diabetes, COPD, steroids, and hypertension. The sample size was too small to perform logistic regression analysis for organ space SSI.

	Univariate Analysis		Multivariate Analysis	
	Odds Ratio (95% CI)	p-value	Odds Ratio (95% CI)	p-value
Minor Complications				
Superficial Surgical Site Infection	1.49 (0.0700 – 15.9)	0.748	7.16 (0.290 – 97.1)	0.144
Urinary Tract infections	1.54 (0.460 – 4.59)	0.456	3.00 (0.630 – 13.7)	0.157
Bleeding Transfusions	3.05 (0.360 – 26.0)	0.273	1.40 (0.130 – 14.8)	0.767
Major Complications				
Readmission	3.00 (0.120 – 76.9)	0.441	1.23 (0.0400 – 35.7)	0.890

**Table 5:** Logistic Regression Analysis for 30-Day Peri and Postoperative Adverse Events Following Anterior and Posterior Colporrhaphy The dependent variable is composite 30-day complications. Controls for multivariate were age, BMI, race (minority or not), ethnicity (Hispanic or not), smoking status, diabetes, COPD, steroids, and hypertension.

	Univariate Analysis		Multivariate Analysis	
	Odds Ratio (95% CI)	p-value	Odds Ratio (95% CI)	p-value
HbA1c ≤ 6.4	Ref	Ref	Ref	Ref
HbA1c ≥ 6.5	2.42 (0.970 – 5.88)	0.052	3.16 (1.00 – 10.2)	0.050

## Discussion

Principal findings

In this study, we aimed to investigate the association between HbA1c levels and complications following anterior or posterior colporrhaphy. Given the lack of data regarding diabetes and its impact on outcomes in this specific surgical procedure, our research fills a crucial gap in the literature. As hypothesized, our analysis revealed that patients with higher HbA1c levels had 3.16 times higher odds of composite complication rate. Notably, UTI emerged as the most common complication. Along with this, there was a notable complication difference between the two HbA1c cohorts within the descriptive statistics. These findings highlight the importance of preoperative glycemic control in patients undergoing colporrhaphy and the potential implications of managing an elevated HbA1c prior to surgical intervention.

In addition to our primary findings regarding the association between HbA1c levels and complications post-colporrhaphy, patients with higher HbA1c levels were older on average (67.9±10.5 vs. 64.1±11.2) compared to those with lower levels. Furthermore, while there were no significant differences in racial distribution between the two cohorts, a higher proportion of patients in the ≥6.5 group had unknown or unreported race. Our analysis also revealed trends toward higher rates of major and minor complications in the ≥6.5 cohort, although these did not reach statistical significance, likely due to the sample size. However, the adjusted analysis confirmed that HbA1c levels ≥6.5 were independently associated with increased odds of composite complications following colporrhaphy, after adjusting for covariates.

Hypertension was significantly more common in the HbA1c ≥6.5 cohort, consistent with clustering of cardiometabolic risk factors in patients with diabetes. Hypertension may contribute to postoperative complications through shared pathways of endothelial dysfunction and microvascular disease, which can impair tissue perfusion and wound healing, and it may also function as a proxy for overall comorbidity burden. We addressed this by adjusting for hypertension in the multivariate modeling.

Results in the context of what is known

These findings are important to consider as the current state of research in pelvic reconstructive surgery, particularly regarding the impact of diabetes and glycemic control, is characterized by a growing recognition of the importance of preoperative risk stratification and optimization [4]. Previous studies have investigated the association between diabetes and surgical outcomes in various urological procedures, such as mesh insertion and prosthesis surgeries, demonstrating higher complication rates among diabetic patients [12,13]. Notably, these studies are performed with surgical implants, resulting in higher rates of infections thought to be due to vaginal flora contamination of mesh, with the most common pathogens being Staphylococcus and Enterococcus [14]. Although colporrhaphy does not include indwelling implants that may act as a nidus for infection, there remained complication rates associated with an increased HbA1c. This is in alignment with critical studies in the field that have shed light on the complex interplay between diabetes and surgical interventions for pelvic floor disorders. Recent findings have elucidated the impact of diabetes on postoperative complications, emphasizing the need for comprehensive risk assessment and management strategies in pelvic reconstructive surgery [15,16]. Systematic reviews highlighted diabetes mellitus as the only factor significantly associated with POP recurrence among various risk factors examined, indicating a need for further research to explore the complex relationship between diabetes and pelvic floor disorders [10]. Despite these advances, gaps remain in our understanding of the specific role of glycemic control in surgical outcomes following colporrhaphy. It is known that pelvic SSIs are caused by native skin flora, predominantly aerobic gram positive cocci, as well as a host of polymicrobial species present in the vaginal microbiota. Documented risk factors for infection in pelvic surgery include HbA1c >6.5 in addition to tobacco use, corticosteroids, and age, to name a few [17]. While infectious organisms may be similar in pelvic procedures with and without mesh use, mechanisms differ and further research is needed to elucidate complications in colporrhaphy specifically. Our study addresses this gap by providing novel insights into the association between increased HbA1c levels and complications specifically in anterior or posterior colporrhaphy. 

As for operative complications, although our regression did not reach significance due to lack of power, our study found that superficial SSI occurred at a higher incidence in the HbA1c ≥6.5 cohort with greater than 7 times the odds. This is relevant as there is a paucity of literature analyzing both SSI and organ space SSI in patients who underwent anterior or posterior colporrhaphy. However, it should be noted that published outcomes of hysterectomy found a 1.6% occurrence of SSI and an occurrence of organ space SSI at 1.1% [18]. Our findings may be explained by the association between elevated HbA1c and higher rates of SSI due to hyperglycemia-associated microvascular complications, delayed wound healing, and impaired immune response [19].

Similarly to operative complications, UTIs were also more common in the HbA1c ≥6.5 cohort as compared to the HbA1c ≤6.4 cohort, paralleling published results suggesting the incidence of UTIs after colporrhaphy was increased [20]. This relationship is further elucidated as elevated HbA1c has been shown to predispose patients to increased rates of UTI due to increased glucose and its promotion of pathogenic bacterial growth adherent to the urothelial tract. Diabetic autonomic neuropathy may further result in impairment of bladder emptying, resulting in increased UTI rates as a result of stasis [21]. 

As for bleeding transfusions, there was no difference in requirement in the HbA1c ≥6.5 cohort as compared to the HbA1c ≤6.4 cohort. Although it has been well established that the presence of diabetes directly causes increased blood viscosity, with blood glucose inversely correlating to survival rates [22]. Furthermore, hyperglycemic stress has been found to result in reduced survival of erythrocytes, resulting in increased red blood cell turnover within other literature [23]. Despite the effect of diabetes on the functionality and structure of erythrocytes, we did not observe a significant difference in requirement for transfusion among the cohorts. Nevertheless, it is imperative to recognize the role of hyperglycemia-induced vascular damage that could play a role in postoperative complications. Further narrowing of accepted HbA1c parameters prior to surgical intervention therefore may result in better predicted outcomes.

Clinical implications

Our significant findings were elucidated by close relevance in research completed in colporrhaphy, hysterectomy, and pelvic reconstruction surgery, suggesting the applicability of our study to a broadened focus [18,20,24]. As our research is specific to anterior and posterior colporrhaphy, it may be difficult to generalize our findings to surgeries outside of the pelvis, although data do indicate that an increased HbA1c is associated with higher adverse outcomes in spinal surgery, cardiac surgery, and major abdominal surgery [9,25,26]. More research should be done in order to assess our specific cutoff of HbA1c ≥6.5 and its association with adverse outcomes in other surgical realms. 

Research implications

Future studies in this field should prioritize several key areas to advance our understanding of the association between diabetes management, as indicated by HbA1c levels, and surgical outcomes in pelvic reconstructive surgery. Additionally, there is a need for prospective studies that can provide more robust evidence on the causal relationship between glycemic control and postoperative complications following colporrhaphy. Future studies should also consider the role of other confounding factors, such as obesity and comorbidities, in mediating the relationship between diabetes and perioperative outcomes. Such efforts will allow informed decision-making on patient candidacy for surgical prolapse repair, improving patient care. 

Strengths and limitations

Our study includes multiple noteworthy strengths. The national database used, NSQIP, contains a large patient cohort, which allows for the application of findings in the future. Our focus was on perioperative complication rates among patient cohorts of HbA1c values above and below a chosen parameter, which has yet to be accomplished in previous studies. However, limitations do remain within our study. Perioperative outcomes are limited to 30 days, preventing long-term outcome assessment. At last, while our analysis is the largest to date assessing perioperative colporrhaphy outcomes and HbA1c, we remain underpowered as only the recent year provided HbA1c as a variable. This may introduce selection bias, limit statistical power, and contribute to the wide confidence intervals and borderline statistical significance observed for the primary outcome, as well as potential type II error for secondary endpoints. In addition, not all patients who underwent anterior or posterior colporrhaphy had a measured HbA1c, shrinking the usable data pool. Because NSQIP records HbA1c only as a preoperative value without detailed timing relative to surgery, variation in the interval between testing and operation may introduce measurement imprecision that could not be fully accounted for in this analysis

## Conclusions

This study provides evidence that higher HbA1c levels are associated with an increased risk of complications following anterior and posterior colporrhaphy. These findings underscore the importance of preoperative glycemic control in optimizing patient outcomes. Establishing clear guidelines for managing diabetes in the perioperative period could lead to improved surgical outcomes for diabetic patients undergoing colporrhaphy.
